# The complete mitochondrial genome of the common strain black carp (*Cyprinus carpio* var*. baisenensis*)

**DOI:** 10.1080/23802359.2020.1869607

**Published:** 2021-02-12

**Authors:** Peipei Wang, Opeoluwa Christiana, Liao Yu, Chengfeng Zhang, Shengyan Su, Yongkai Tang

**Affiliations:** a Key Laboratory of Genetic Breeding and Aquaculture Biology of Freshwater Fishes, Ministry of Agriculture; Freshwater Fisheries research Center, Chinese academy of Fishery Sciences, Wuxi, PR China; b Wuxi Fisheries College, Nanjing Agricultural University, Wuxi, PR China; c Guangxi Fisheries Introduction and Cultivation Center, Nanning, PR China

**Keywords:** Mitochondrial genome, phylogenetic, Cyprinus carpio var. baisenensis, integrated rice-agriculture

## Abstract

The common strain black carp (*Cyprinus carpio* var*. baisenensis*), known for its black skin is cultured in the integrated rice-agriculture system and non-escape property under torrential floods. The total mitogenome length of *Cyprinus carpio* var. *baisenensis* obtained in this study was 16,478bp, consisting of 13 protein-coding genes, 22 tRNA genes, 2rRNA genes (large and small), a light strand origin of replication, and one major non-coding region. By providing the complete mitochondrial genomes of *Cyprinus carpio* var. *baisenensis,* we will further understand the phylogenetic relationships within genus.

## Introduction

The common carp (*Cyprinus carpio*) is a widespread freshwater fish originated in Eurasia. Over the years, the common carp has become one of the most important edible and popular aquarium fish, comprising a large number of strains worldwide because of its cultural history and domestication (Zhou et al. 2003). Common carp populations have the highest numbers of domesticated strains in china compared to other countries worldwide. These renowned strains include Huanghe carp (HH) Genome Wide Identified Main QTL Related to Growth Performance of Huanghe Carp New Strain (*Cyprinus carpio hacmalopterus*), Oujiang color carp (OJ), Hebao red carp (HB), Songpu mirror carp (SP), Xingguo red carp (XC) and Genome Wide Identified Main QTL Related to Growth Performances of Huanghe Carp New Strain (*Cyprinus carpio hacmalopterus* Temminck *et* Schlegel) (Dong et al. 2015; Su et al. 2018).

The common strain black carp (*Cyprinus carpio* var*. baisenensis)* from Guangxi has a history with the local Bourau people who prefer the black color. The sub-species color reflects the interaction between Bourau and their surrounding environment. This species is selected, geographically isolated and breeded, so there is no germplasm pollution. The common strain black carp is cultured in an integrated rice culture. This culture system helps the individuals to persist when there are floods. Supplementary Figure S1 shows the environment of the black carp and the pictures of the black carp. Complete mitochondrial genome sequencing can provide useful information for species conservation and identification (Li et al. 2016). Complete sequencing of mitochondrial genome also plays an important role in the study of *Cyprinus carpio* var*. baisenensis* phylogeography and genetic diversity of the population (Guan et al. 2018). mtDNA sequences analysis is one of the most effective methods available for population studies because it is simple and allows analyzing a large numbers of samples (Hilbish 1996; Nishida 1998). The common strain black carp was obtained from Guangxi. In this study, the complete mitochondrial genome of *Cyprinus* carpio var*. baisenensis* was sequenced using the Next-generation sequencing technology on Illumina NovaSeq. The assemble software used is GetOrganelle v1.6.2e (Jin et al. 2020). This annotation method was conducted using MITOS Webserver (Bernt et al. 2013). Total genomic DNA was extracted from the muscle of *Cyprinus carpio var.baisenensis* using a DNA extraction kit (Simgen, China) following the manufacturer’s instructions. Twelve primer sets were used in the PCR amplification, and the amplification was carried out in a total volume of 25 ml including: 60 ng total genomic DNA, 1 U Ex-Taq DNA polymerase, 2.0 mM MgCl2, 2 mM dNTP, and 10pM of each primers. The total mitogenome length of the common strain black carp obtained in this study was 16,478bp in the, which was deposited in GenBank with the accession number MT780875, under the Biosample number of SAMN16679851, Bioproject ID of PRJNA674914, SRA of SRR12996635 and SRP 291369 in NCBL (https://www.ncbi.nlm.nih.gov/). The mitogenomes of *Cyprinus carpio* var*. baisenensis* consisted of 13 protein coding genes, 22 tRNA genes, 2rRNA genes (large and small), origin of light (O_L_) strand of replication and one major non-coding region (D loop) (Table 1). The *Cyprinus carpio* var*. baisenensis* sequence protein coding total length is 11,415bp and most of these proteins and tRNA genes are encoded on the D-strand (Direct strand).). The total length of all tRNA genes was 1,563bp and rRNA total length was 2,586bp. All the PCGs start with ATG codon expect for COX1 that starts with GTC (Table 1). Seven protein coding genes have TAA as stop codon (NAD1, COX1, ATP6, COX3, NAD4L, NAD5, NAD6) while NAD2, ATP8, NAD3 have TAG as stop codon. O_L_, COX2, NAD4 and COB have AAA, CCT, ATT and CTT, respectively, as their stop codon (Table 1).

**Table 1.** Mitochondrial genome characteristics of *Cyprinus carpio var.baisenensis*.

	Position			Codon					
Genes	From	To	Size of nucleotide (bp)	Start	Stop	Amino acid	Anti-codon	Intergenic nucleotide	Strand
tRNA^Phe^	1	69	69	–	–	–	GAA	0	D
rRNA	70	1022	953	CAA	AAA	316	–	0	D
tRNA^Val^	1025	1096	72	–	–	–	TAC	0	D
rRNA	1119	2751	1633	TTA	AAT	543	–	0	D
tRNA^Leu^	2776	2851	76	–	–	–	TAA	0	D
NAD1	2853	3827	975	ATG	TAA	324	–	4	D
tRNA^Ile^	3832	3903	72	–	–	–	GAT	–2	D
tRNA^Gln^	3902	3972	71	–	–	–	TTG	1	R
tRNA^Met^	3975	4043	69	–	–	–	CAT	0	D
NAD2	4044	5090	1047	ATG	TAG	348	–	0	D
tRNA^Trp^	5089	5159	71	–	–	–	TCA	2	D
tRNA^Ala^	5162	5230	69	–	–	–	TGC	1	R
tRNA^Asn^	5232	5304	73	–	–	–	GTT	0	R
O_L_	5307	5338	32	TTT	AAA	28	–	–2	D
tRNA^Cys^	5338	5404	67	–	–	–	GCA	1	R
tRNA^Tyr^	5404	5474	71	–	–	–	GTA	1	R
COX1	5476	7026	1551	GTG	TAA	516	–	0	D
tRNA^Ser^	7027	7097	71	–	–	–	TGA	3	R
tRNA^Asp^	7101	77172	72	–	–	–	GTC	13	D
COX2	7186	77876	691	ATG	CCT	229	–	0	D
tRNA ^Lys^	7877	7952	76	–	–	–	TTT	1	D
ATP8	7954	8118	165	ATG	TAG	54	–	–7	D
ATP6	8112	8795	684	ATG	TAA	227	–	–1	D
COX3	8795	9580	786	ATG	TAA	261	–	0	D
tRNA^Gly^	9580	9651	72	–	–	–	TCC	0	D
NAD3	9652	10,002	351	ATG	TAG	116	–	0	D
tRNA^Arg^	10,001	10,070	70	–	–	–	TCG	0	D
NAD4L	10,071	10,367	297	ATG	TAA	98	–	–7	D
NAD4	10,361	11,741	1381	ATG	ATT	459	–	0	D
tRNA^His^	11,742	11,810	69	–	–	–	GTG	–1	D
tRNA^Ser^	11,811	11,879	69	–	–	–	GCT	2	D
tRNA^Leu^	11,881	11,953	73	–	–	–	TAG	3	D
NAD5	11,957	13,780	1824	ATG	TAA	607	–	–4	D
NAD6	13,777	14,298	522	ATG	TAA	173	–	0	R
tRNA^Glu^	14,299	14,367	69	–	–	–	TTC	4	R
COB	14,373	15,513	1141	ATG	CTT	379	–	0	D
tRNA^Thr^	15,514	15,585	72	–	–	–	TGT	–1	D
tRNA^Pro^	15,585	15,654	70	–	–	–	TGG	0	R
D loop	15,673	16,478	806	–	–	–	–	0	D

The length of 22 tRNA genes varies from 67 bp (tRNA^Cys^) to 76 bp (tRNA^Leu^ and tRNA^Lys^). The two rRNA genes have nucleotide size of 163 bp respectively and are located in a position between tRNA^Phe^ and tRNA^Leu^, and separated by tRNA^Val^. The L-strand origin of replication (O_L_), it is located identified between the tRNA^Asn^ and tRNA^Cys^ and has a size of 32 bp. The control region (D-loop) with the nucleotides size of 806 bp is located between tRNA^Pro^ and tRNA^Phe^ (Table 1).

**Figure 1.**
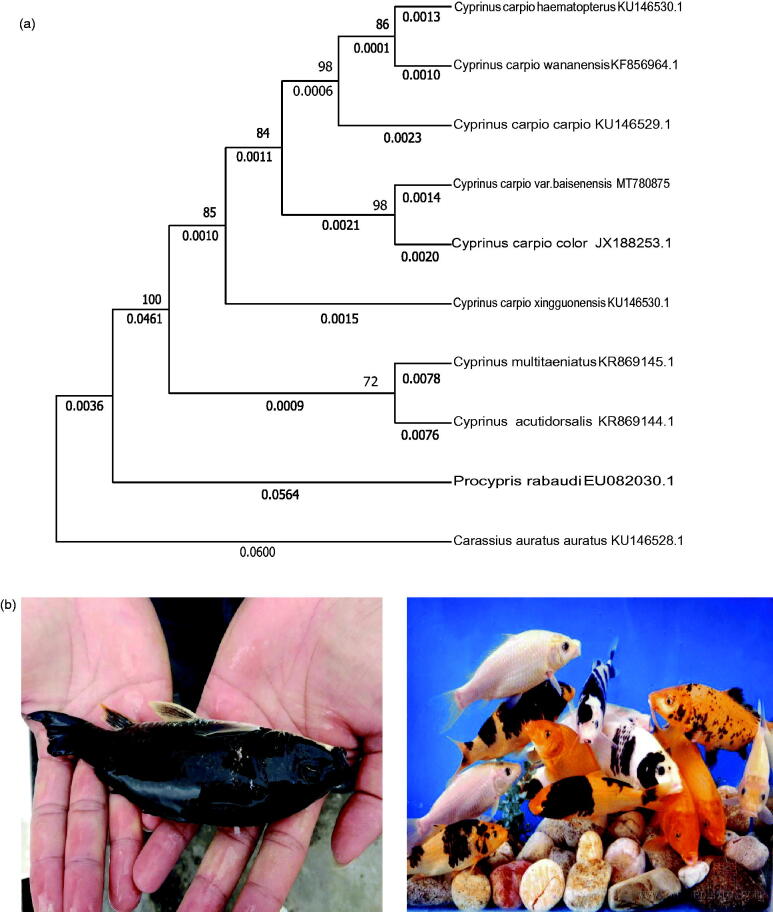
(a) The maximum likelihood (ML) phylogenetic tree of Cyprinus carpio var.baisenensis and related species. Numbers on each node are bootstrap probability. The number after the species name is the GenBank accession number.

The phylogenetic position of *Cyprinus carpio* var*. baisenensis* was reconstructed based on the complete mitogenomes of11 species using maximum-likelihood (ML) methods and the MEGA X (Kumar et al. 2004). The phylogenetic tree of the common strain black carp shows that *Cyprinus carpio* var*. baisenensis* is the sister species of *Cyprinus carpio color* (Figure 1(a)).

## Data Availability

The genome sequence data that support the findings of (Complete Mitochondrial Genomes (mtDNA) of Common Strain Black Carp **(***Cyprinus carpio var.baisenensis*) are openly available in GenBank of NCBI at (https://www.ncbi.nlm.nih.gov/) under the accession no. MT780875. The associated Bio-Project, SRR, SRP and Bio-Sample numbers are PRJNA674914, SRR12996635, SRP 291369, and SAMN16679851 respectively.
